# Comparison of Stresses Induced by Fiber Post, Parapost and Casting Post in Root Canals by Photoelasticity Method

**Published:** 2010-02-20

**Authors:** Aliakbar Rezaei, Fereidoon Soltani, Fariborz Vafaei, Masoumeh Khoshhal, Majid Reza Ayatollahi, Naser Soltani, Morteza Nejati

**Affiliations:** 1. Department of Prosthodontics, Dental School, Shahid Beheshti University of Medical Sciences, Tehran, Iran.; 2. Department of Prosthodontics, Dental School, Hamadan University of Medical Sciences, Hamadan, Iran.; 3. Department of Periodontics, Dental School, Hamadan University of Medical Sciences, Hamadan, Iran.; 4. Department of Mechanical Engineering, Iran University of Science and Technology, Tehran, Iran.; 5. Department of Mechanical Engineering, Tehran University of Medical Sciences, Tehran, Iran.

**Keywords:** Casting Post, Endodontic Post, Fiber Post, Parapost, Root Canals, Stress Analysis

## Abstract

**INTRODUCTION:**

Many studies have been performed to evaluate the stress distribution around endodontic posts; those which compared posts composed of different materials are rare. The aim of this study was to compare stresses induced in dentin by three structurally different posts using photoelasticity method.

**MATERIALS AND METHODS:**

Nine blocks of PSM-5 Photoelastic material with 45×45×10 mm dimension were prepared. In each block, a canal 9 mm in length and 0.8 mm in width was drilled. Blocks were divided into 3 groups of three each. In the first group, the canals were prepared for insertion of Fiber Post with 1.25 mm width. In the second group, the canals were prepared for insertion of ParaPost with 1.25 mm width and the canals in the third group were prepared for casting post similar to the above samples. Casting Post pattern was made by Duralay resin and casted by Ni-Cr alloy. All posts were cemented in canals with Panavia cement. The stresses were evaluated in the polariscope under three different conditions: 1) without load, 2) with 135 N vertical load, and 3) with 90 N oblique load (26° inclination to post long axis). The fringe orders in the cervical, middle and apical regions of the posts were evaluated and compared with each other.

**RESULTS:**

Application of the vertical load induced a high stress concentration (FO=4) in the apical region of the ParaPost, while lower stress was observed in the middle (FO=2) and cervical region (FO=2+). Fiber Post and Casting Post showed even stress distribution (FO=2+). High stress concentration was detected with the application of oblique force in the cervical region of ParaPost (FO=5) and Casting Post (FO=3+). Fiber Posts fractured before reaching 90-N loading force.

**CONCLUSION:**

The stress distribution around Fiber Post and Casting Post were constant in comparison with ParaPost. Fiber Post with 1.25 mm width was not recommended in situations with high oblique stresses.

## INTRODUCTION

Prosthetic restoration of endodontically treated teeth is a great challenge in dentistry [1]. Lost tooth structures are primarily substituted by an alloplastic material to provide a preparation with an adequate surface area to retain a prosthetic crown or bridge. If the remaining tooth structure is inadequate for retention of a direct core build-up material, a post must be used to retain the core [2].

Custom-fabricated cast post and cores has been advocated as the gold standard restoration for decades [3]. In recent years, new methods with regard to biologic principles and structural compatibility between restorative material and tooth structure have gained more attention. This concept in treatment consists of using materials which are reinforced by fiber and use of adhesive resins [4].

Several factors should be considered when selecting a post system such as the design and material of their post, and their effect on stress distribution in dentin. If the induced stresses become exceedingly high, fracture of the tooth structure may occur [1].

It is believed that Fiber Posts bend under masticatory forces, leading to the distribution of stresses between post and tooth structure. Rigid metal posts resist lateral forces without distortion and this results in stress transfer to the less rigid dentine, which increases the potential for root cracks and fractures [5].

Photoelasticity is a useful technique for evaluating the stresses responsible for failure of a structure, especially one with irregular form [6]. This technique is a relatively qualitative visual measurement based on the ability of certain transparent materials to exhibit interference fringes when stressed in a field of polarized light. The distinct fringes illustrate zones of stress intensity and concentration with a sequence of colored bands [6]. This technique has been used in several dental studies to analyze the stress distribution of various posts [1][6][7][8][9][10][11][12][13][14][15][16][17][18][19].

In 1972, Standlee et al. examined stress interaction between endodontic posts and their supporting structures [18]. This was the first endodontic study using photoelastic stress analysis [6]. They concluded that smooth-sided parallel posts generate high apical stress; tapered posts exhibit a wedging effect and produce the highest shoulder stress concentrations. Therefore the clinical axiom that post length should approximate anatomic crown length for optimal distribution of stress appears to be true.

In 1982, Mattison applied photoelastic method to investigate the stress distribution around casting gold endodontic posts with 0.05 and 0.07 inches width. With an increase in the post width and applied load, stresses increased in dentin [6].

Cohen et al. investigated the stress distribution around four groups of prefabricated posts: Flexi Post, Flexi Flange, ParaPost and Access Post. In all groups, minimal stresses were observed after cementation and before loading. Under a vertical load of 134 N, even and symmetric stress patterns were observed around Access Post, Flexi Flange and Flexi Post. The stress patterns recorded for ParaPost by 134 N vertical loading were symmetric with more stress concentration in the apical region than coronal; and for oblique loading were asymmetric with more concentration along apical surface of the post [7].

Kishen et al. study comprised two parts: a photoelastic study and a fractographic analysis. They compared stress patterns induced in the photoelastic study with the plane of fracture in the fractographic analysis using mandibular incisors restored with ParaPost and composite core. A strong correlation was found between the photoelastic stress patterns recorded and the plane of fracture observed by SEM study [8].

Although several studies evaluate stress distribution around different posts, only a few studies have compared stresses around posts with different materials. The aim of this experimental study was to analyze stress distribution around three commonly used dental posts by the photoelastic method.

## MATERIALS AND METHODS

Nine blocks of PSM-5 photoelastic material (Measurements Group Inc., Raleigh, NC, USA) were prepared with 10×45×45 mm dimensions. PSM-5 is an epoxy resin with high elastic modulus that is similar to dentine, good stress-optic and creep properties [11]. The prepared blocks were tested by a polariscop (Photolastic inc., Raleigh, USA) to make sure they were free of residual stresses.

A canal 9 mm in length and 0.8 mm in width was prepared in each block by a press drill instrument (Superstar Co, China) with vertical angulation to the edge of blocks.

The blocks were divided into 3 groups with 3 samples each; group 1 was considered for restoration with FiberLux Fiber Post (Coltène/Whaledent, USA), group 2 for restoration with Para Post (Coltène/Whaledent, USA) and group 3 for restoration with custom made Casting Post.

Para Post and Fiber Post used in this study were parallel-sided and flat-ended and had the same width of 1.25 mm. The canal preparation for prefabricated posts was performed according to the manufacturer instructions.

Preparation for the Cast Posts was similar to prefabricated post canals. Casting Post was made with Ni-Cr alloy (Dentsply Sankin, Tokyo, Japan) with the same width and design as the prefabricated posts.

Before cementation, all the blocks were tested by polariscope to avoid the presence of any residual stress.

Posts were cemented in the canals by Panavia F cement (Kuraray Dental, Japan) according to manufacturer’s instructions. The post lengths were reduced in such a way so that 4 mm of supra canal structure remained.

All specimens were tested by polariscope and photographed with and without color filter in three loading conditions:

- Without loading

- With 135-N vertical loading

- With 90-N oblique loading making 26˚ angle to the long axis of post.

The photographs were analyzed and the fringe orders were determined in three equal segments; coronal, middle and apical thirds.

**Figure 1:**
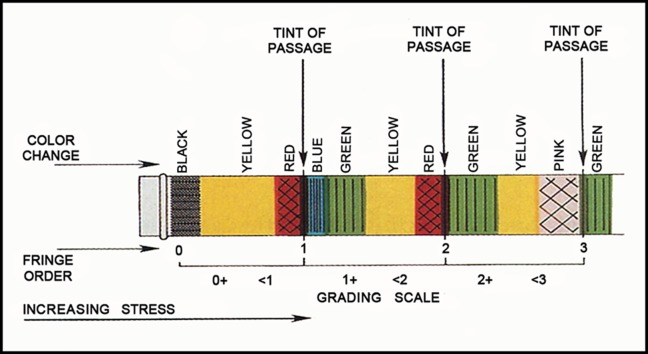
Illustration of fringe orders and color changes in photoelastic stress analysis [11]

In order to determine the fringe order, the pattern illustrated in Figure 1 was used [11]. The difference value in fringe order which was equal to or greater than one was assumed to be significant.

## RESULTS

In the absence of loading, all specimens were stress free. In groups where the same post and similar loading conditions were kept, the stress patterns were identical.

**Figure 2:**
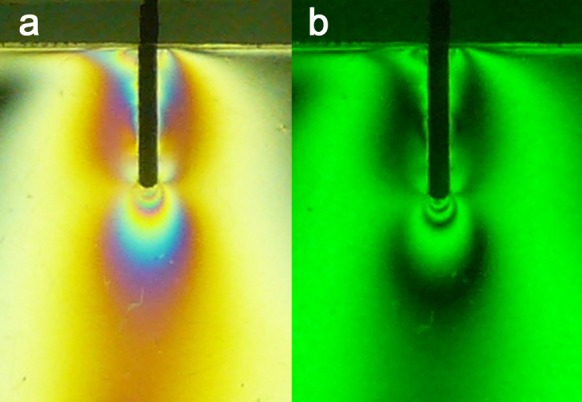
Isochromatic fringe patterns of the Parapost subjected to vertical loading a) colored, b) monochromatic

**Figure 3:**
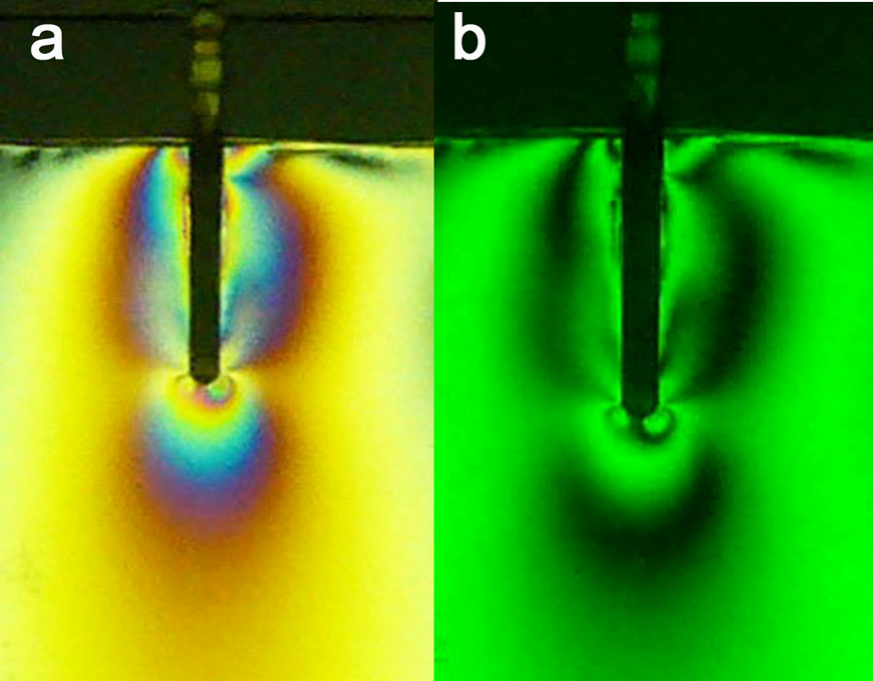
Isochromatic fringe patterns of the Fiberpost subjected to vertical loading a) colored, b) monochromatic

**Figure 4:**
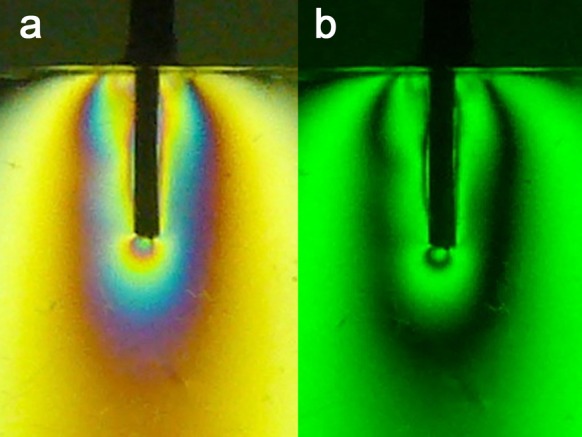
Isochromatic fringe patterns of the Casting post subjected to vertical loading a) colored, b) monochromatic

Under 135-N vertical loading, the following findings were recorded:

1) ParaPost: the stresses were concentrated in the apical area (Fringe Order (FO) = 4). The coronal and middle regions had moderate stresses (FO = 2+) (Figure 2).

2) Fiber Posts: the stresses were evenly distributed along the post (FO = 2+). Local stress concentrations in the apical and coronal regions were observed (Figure 3).

3) Casting Post: the stresses were evenly and symmetrically distributed along the post (FO = 2+) (Figure 4).

**Figure 5:**
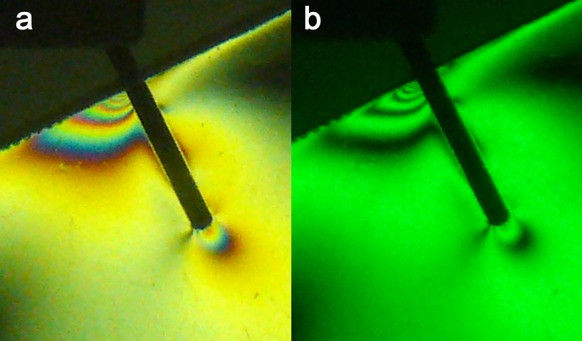
Isochromatic fringe patterns of the Parapost subjected to oblique loading a) colored, b) monochromatic

Under oblique loading, the following findings were recorded:

1) ParaPost: higher stress concentration was recorded in the coronal region opposite to the loading side (FO = 5). In the middle (FO = 1) and apical (FO = 2) regions lower stresses were observed (Figure 5).

2) Fiber Post: this post fractured before reaching 90-N load; hence stress analysis was not performed.

3) Casting Post: higher stresses were concentrated in the coronal region opposite to the load side (FO = 3+).

**Figure 6:**
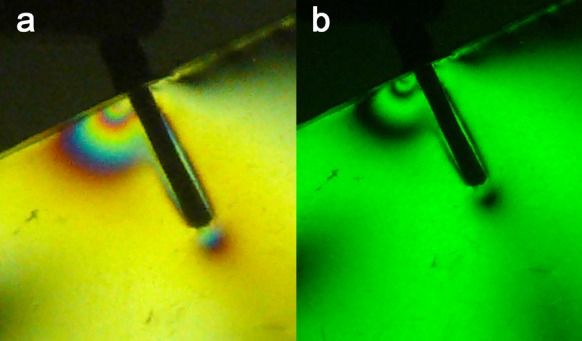
Isochromatic fringe patterns of the casting post subjected to oblique loading a) colored, b) monochromatic

The lower stresses were recorded in the middle and apical regions (FO = 1+) with even and symmetric distribution (Figure 6).

## DISCUSSION

The Photoelastic material used in this study, PSM-5, has a similar modulus of elasticity to dentin. This material has been previously utilized for stress analysis in dentin in several photoelastic studies [6][11][12][13][14][15][16][17]. Non-anatomic modeling was used in this study; thus variables such as the root morphology, the crown and periodontal membrane preparation had no effect on the results [18]. This type of modeling has been used in several previous studies [1][7][11][12][14][16][19]. Photoelastic studies have some limitations; Kishen et al. demonstrated a strong correlation between the stress concentration recorded in the photoelastic study and the plane of fracture observed in the fractographic study [8]. Bearing this in mind, the results of this study can be used for predicting the areas of stress concentration and the possibility of tooth fracture in clinical situations.

After cementation and before loading, all specimens were stress free. This finding is in correlation with Cohen et al. [9] and Caputo et al. [16]. Burns et al. reported low stress concentration as FO = 0.5 [11]; and Cohen et al. reported “minimal stresses” after cementation [7].

When Para Post underwent vertical loading, they demonstrated high stress concentration around the apical region. Lower stresses were observed in the middle and coronal parts. The stress patterns were symmetric. This pattern correlated with Cooney et al. [19], Burns et al. [11] and Cohen et al. [7].

With oblique loading, a high stress concentration was recorded around the coronal region of ParaPost. The stresses in the middle and apical regions were lower. Again this concurs with Cooney et al. [19] and Burns et al. [11]. However, Cohen et al. [7] reported higher stresses in the apical region than coronal.

Difference in the results of Cohen’s study and other studies can be due to the various methods used in cavity preparation. In Cohen’s study, endodontic drills were used to prepare the canals; however they are not suitable instruments for producing holes in photoelastic materials and their use may lead to residual stresses. The results of their study confirm the presence of stresses in the specimens before loading. These stresses can affect the final result.

In vertical loading on the casting post, the stress patterns were evenly distributed along the post, with FO=2. This finding is in conflict with Mattison study in 1982 [6] and Mattison and von Fraunhofer study in 1983 [13] as well as other studies [6][13]. In Mattison’s first study a mean of FO=5 was observed; in his second study a mean FO=4.3 under vertical loading was reported. The difference between our results and the Mattison’s study can be due to the different post materials used i.e type III Gold and Ni-Cr in the present study. Another explanation is the difference in intra-canal post length which was 9 mm in the present study and undeclared in Mattison’s study.

Vertical loading on Fiber Post caused symmetric and even distribution of stresses along the post with a value of FO=2+. No photoelastic study was found on Fiber Posts which could be compared with this study.

Fiber Posts fractured before reaching 90 N oblique loading. This concurs with Teixeira et al. study [20] where four types of Fiber Posts with similar widths to the Fiber Post in the present study were used (D.T. Light-Post, FiberKleer Tapered Post, FiberKleer Parallel Post and FibreK). They were loaded by a 45 degree oblique load until they reached fracture point. The mean fracture strengths were between 45-72 N for the different posts. These findings confirm that glass Fiber Posts with 1.25 mm width do not have enough strength to tolerate 90 N oblique loading.

## CONCLUSION

Under vertical loading, stresses were evenly distributed around Fiber Post and Casting Post. The stress was mainly concentrated in the apical area in ParaPost system.

Under oblique loading, stress became concentrated around the coronal area of ParaPost and Casting Post; in the ParaPost system, stresses were even higher.

The use of glass fiber posts with 1.25 mm width in cases with high oblique loading is not recommended.
